# Migration status and internet use: gender differences in mental health among rural older adults in China

**DOI:** 10.3389/fpubh.2026.1697446

**Published:** 2026-02-23

**Authors:** Xiaoxiao Li, Taixiang Duan, Sizhan Cui, Dongyang Zhou, Yaping Hu, Shuijing Xu

**Affiliations:** 1 Institute of Population Research, Nanjing University of Posts and Telecommunications, Nanjing, China; 2 Jiangsu High-Quality Development Comprehensive Evaluation Research Base, Nanjing University of Posts and Telecommunications, Nanjing, China; 3 Law school, Southwestern Petroleum University, Chengdu, China; 4 Department of Sociology, University of Oxford, Oxford, United Kingdom; 5 School of Public Management and Law, Anhui University of Technology, Ma'anshan, China; 6 School of Education and Science and Technology, Nanjing University of Posts and Telecommunications, Nanjing, China

**Keywords:** gender differences, internet use, mental health, migration status, rural older adults

## Abstract

**Background:**

A study was conducted to examine the impact of Internet use on the mental health of male and female older adults with different migration statuses living in rural China.

**Methods:**

Data came from the China Family Panel Studies (CFPS) 2020. The propensity score matching method was adopted to analyze the effect of Internet use on mental health of rural older adults as a function of migration status and gender.

**Results:**

Our findings indicated a progressive decline in depressive symptoms and a corresponding increase in mental well-being among older migrants, left-behind older adults, and those without migrant children. Furthermore, the mental health benefits associated with Internet use increased alongside this improvement. Importantly, older migrant women derived fewer mental health benefits from Internet use than their male counterparts, as did left-behind older women relative to men in the same cohort.

**Conclusion:**

Since the mental health of the older migrants was worse than that of left-behind older adults or those without migrant children, and older migrant women derived fewer mental health benefits from Internet use than their male counterparts, it is necessary to pay more attention to rural older migrants, especially older women, to improve their willingness and ability to use the Internet.

## 1 Introduction

Population aging represents one of the most intricate social challenges facing numerous countries today, including China. By 2050, the population aged 65 and above is projected to reach 400 million, or 26.9% of the total population. This places China among the countries with the highest proportion of older population in the world (1). As the population has aged, improving older adults' mental health has emerged as a critical task for enhancing their quality of life. At the same time, the relaxation of China's household registration (Hukou) system has facilitated increased rural-to-urban migration. As of 2024, approximately 132 million rural laborers were employed in urban areas (2). In traditional Chinese society, particularly in rural areas, the family has long served as the primary unit of older adult care, with adult children typically co-residing with their parents and assuming primary caregiving responsibilities (3). However, with large-scale labor migration to urban areas, many rural older adults have become either left-behind or migrant older adults due to their adult children's migration for work. This transition has been associated with a gradual decline in traditional family support, significantly weakening rural older adults' social capital (4), with negative implications for their mental health (5, 6). Studies have shown that depressive symptoms are more prevalent among older adults in rural China than among other older populations (7). Consequently, against the backdrop of population aging and population mobility, improving the mental health of rural older adults has become a key priority in responding to the challenges of an aging society.

At the same time, rapid progress in China's Internet infrastructure has significantly increased Internet and smartphone usage among rural residents. As of June 2025, the Internet penetration rate in rural China reached 69.2% (8). Internet use has substantially expanded patterns of intergenerational interaction and avenues for accessing social capital among rural older adults, which is associated with improved mental health outcomes (9). The Internet has become an essential tool for left-behind older adults to stay connected with migrant family members, as well as for migrant older adults to adapt to new environments while maintaining ties with relatives and friends in their places of origin.

However, research examining mental health among older adults with different migration statuses has predominantly focused on either left-behind or migrant older adults (10–13). These studies have often compared older migrants with local residents in urban areas or made distinctions within the older migrant group itself, seldom integrating both left-behind and migrant older adults into a cohesive analytical framework. Even fewer studies have compared left-behind older adults, older migrants, and older adults without migrant children (14). Finally, research exploring these three groups from the perspectives of gender and Internet use remains particularly limited. Furthermore, research on gender differences in the impact of Internet use on the well-being of older adults has produced inconclusive results (3, 15, 16). This may be attributable to the insufficient consideration of urban–rural differences in prior research, as well as gender differences in access to social capital through Internet use among rural older adults under different migration statuses. Accordingly, we conducted a study aimed at addressing these research gaps. Drawing on the theoretical framework of social capital, this study examines the effects of Internet use on the mental health of rural older adults across different migration statuses, and assesses the existence and nature of gender differences.

The concept of social capital was first systematically articulated by the French sociologist Pierre Bourdieu (17), who argued that its core lies in social networks and relationships (18). Ellison likewise emphasized that interpersonal relationships and norms of reciprocity constitute the central elements of social capital (19). Despite its widespread use, social capital remains a contested concept, with no universally accepted definition or standardized method of measurement (20). In the context of public health research, trust, social networks, and social participation are commonly regarded as key indicators of social capital (21). Trust is grounded in value judgments and forms the basis for reciprocal cooperation in social interactions. Social networks are the interactions and connections individuals have or establish with one another. Social participation denotes the stable system formed through social interactions and engagement in various formal or informal activities (22). Research has shown that social capital has a significant impact on individuals' mental health, including that of migrants and older adults (23, 24), the populations examined in this study.

Based on relationships' strength and the types of resources provided by them, social capital can be further categorized into bonding social capital and bridging social capital (21). Generally, bonding social capital refers to strong ties and emotionally significant relationships within homogeneous groups, such as relatives, close friends, and neighbors (19). Bridging social capital is primarily obtained through weak ties, less frequent social interactions, and relationships characterized by lower levels of emotional intimacy (25). This form of social capital provides individuals with instrumental resources, such as information and knowledge (26). Through both strong-tie and weak-tie networks, individuals can access not only tangible resources but also intangible benefits, including mutual trust, emotional support, and friendship (27). However, the relative importance of different forms of social capital varies across social groups and contexts (28). Studies have found that, compared with older non-migrants, older migrants rely more heavily on close-knit, homogeneous social networks to obtain socioemotional support and assistance, and their bonding social capital is positively associated with mental well-being (29). In the Chinese context, strong intergenerational ties and co-residence with adult children contribute to better mental health among older adults (30). In rural China, family relationships, particularly ties with adult children, may be more critical for older adults' mental health than other types of social connections. However, as adult children migrate for work, left-behind older parents experience increased physical distance from their children, which may weaken intergenerational ties compared with those whose children have not migrated. Moreover, many left-behind older adults face the dual burden of caring for grandchildren and managing agricultural work, which increases their vulnerability to mental health problems (14). In particular, depressive symptoms tend to worsen as the duration of their children's migration lengthens (31). When older migrants relocate from their hometowns to relatively unfamiliar environments, their participation in community activities is often limited (32), leading to diminished social capital (33). This reduction in social capital adversely affects their mental and physical health (5, 34).

However, the scope of social capital has been successfully extended into the online sphere, highlighting the positive effects of various forms of online communication (35). Internet technologies help mitigate the negative impact of adult children's migration on the social support of left-behind older adults and significantly reduce depressive symptoms among this group (10). They also enable older migrants to maintain close ties with family members and friends in their places of origin while expanding their social networks (36), thereby compensating for the loss of offline close friendships and contributing to higher life satisfaction and better mental health (37, 38).

Moderate Internet use has been shown to improve older adults' mental health (39, 40), and frequency of Internet use is positively related to the mental health of rural older adults (41). However, older people, especially those with lower socioeconomic status, continue to face substantial challenges in adopting information and communication technologies (ICTs). As a result, they are more likely to be excluded from the digital society (42).

In addition, compared to other age groups within the migrant population, older migrants face greater challenges in adapting to life in their new environments, integrating into local communities, and establishing new social networks. Although left-behind older adults may experience a decline in family support, their networks of other relatives and friends in their hometown often remain intact. Moreover, they may have the opportunity to cultivate broader friendships due to the absence of their children (43), thereby avoiding the need to adapt to a new social environment. Therefore, from the perspective of social capital theory, the Internet serves to amplify social capital for non-migrant older adults living with their children, maintain it for left-behind older adults, and reconstruct it for older migrants. As a result of their low level of social capital when they initially migrate, older migrants face the most difficulties in using the Internet to navigate the challenges associated with migration. Left-behind older adults experience slightly fewer obstacles when using the Internet to cope with the implications of their children's migration. The positive impact of Internet use on the mental health of rural older adults may be minimal among older migrants and most pronounced among those without migrant children.

The impact of Internet use on the mental health of rural older adults may also vary by gender. Research conducted in China has suggested that the relationship between social capital and mental health is more pronounced among older women than among their male counterparts (44). This may be related to the types of social networks individuals are embedded in. Providing social support among network members is one of the most important functions of social networks (45). There are different types and sources of social support, each exerting distinct effects on mental health (46). Social support is generally classified into three categories: emotional support (“expressions of sympathy, love, trust, and care”), instrumental support (“tangible assistance and services”), and informational support (“suggestions and information”) (45). Traditional male-dominated social norms impose gendered role expectations that encourage women to prioritize close relationships, making them more likely to seek emotional support (47) and accumulate bonding social capital. In regions with more traditional gender role attitudes, women's social networks tend to include a higher proportion of children and spouses, and have emotionally closer networks, than men (48). The loss of children or grandchildren is more likely to undermine women's mental health than men's (49). Moreover, sibling relationships among older adults, particularly sisterhood ties, become deeper, closer, and more important in later life (50). Research shows that formal ties, such as involvement in community organizations, have a stronger impact on life satisfaction among older men (51), whereas informal ties with family members and friends may play a more important role in shaping the well-being of older women. This pattern is even more pronounced in rural areas than in urban settings, where gender-role attitudes tend to be more modern. This implies that although being left behind or migrating may adversely affect older adults' mental health, older women are more likely to compensate for these negative effects through bonding social capital or emotional support available compared with men in their hometowns. However, older women with lower social status and limited technological experience may grapple with feelings of low self-esteem and anxiety when using ICT (52). In contrast, men are more likely than women to obtain various forms of social capital through the Internet in destination areas, build local social ties, and acquire bridging social capital and instrumental social support. Studies also indicate that older migrant men demonstrate higher levels of urban adaptation than their female counterparts (53). Therefore, the positive impact of Internet use on the mental health of left-behind older women may be less pronounced than that experienced by their male counterparts.

Based on the above theoretical and empirical considerations, we propose the following hypotheses:

**H1:** the positive effect of Internet use on the mental health of rural older adults will be greater for older adults without migrant children than for left-behind older adults, and in turn greater for them than for older migrants.

**H2:** the positive impact of Internet use on the mental health of left-behind older women will be less than for left-behind older men.

**H3:** the positive impact of Internet use on the mental health of older migrant women will be less than for older migrant men.

## 2 Data, measurement, and methods

### 2.1 Data

The sample data for this study were derived from the 2020 China Family Panel Studies (CFPS2020). Administered by the Social Science Survey Center at Peking University, the CFPS investigated both the economic and non-economic welfare of Chinese residents, encompassing a wide array of research topics such as economic activities, educational attainment, family relationships and dynamics, population migration, and physical and mental health. The survey targets households and eligible family members who meet the project's access criteria in 25 provinces, municipalities, and autonomous regions across China (54). The CFPS 2020 is the sixth national survey conducted as part of this initiative. Given that we explored the impact of adult children's migration on the psychological well-being of rural older adults, it is noteworthy that the age range of rural parents affected by this migration is relatively broad. Consequently, to align with the research objectives, our analysis focused specifically on rural individuals aged 50 and above. The final sample for our analysis comprised 6,408 respondents. Given the relatively low proportion of missing data, observations with missing values on key variables were excluded from the analytical sample. No imputation procedures were applied.

### 2.2 Measurement

#### 2.2.1 Dependent variable

In this study, the dependent variable was operationalized using the 20-item Chinese version of the Center for Epidemiologic Studies Depression Scale (CES-D 20), which was included in the CFPS 2020 dataset and was designed to evaluate depressive symptoms in adult respondents. Scores on this scale range from 0 to 72, with higher scores indicating more severe depressive symptoms and worse mental health status.

#### 2.2.2 Independent variable

This study focuses on differences among left-behind older adults, migrant older adults, and rural older adults whose children have not migrated. The migration status of these three groups is defined according to both older adults' own migration status and their children's migration status. The CFPS asked respondents to report household members who had worked away from home to earn income during the past year. Respondents were also asked about their current place of household registration (Hukou), with response options including: (1) the same village/residential community as the current residence, (2) another village/residential community within the same township/subdistrict, (3) another township/subdistrict within the same county/city, (4) another county/city within the same city/district, (5) another city/district within the same province, or (6) another province within China. Response categories (1)–(3) indicate that the place of household registration and the place of residence are located within the same county/city. Based on these two questions and the Chinese context, the three types of older adults are defined as follows: (1) Older adults with no child migration: individuals whose place of household registration and place of residence are located within the same county/city and who had no child working away from home during the past year. (2) Left-behind older adults: individuals whose place of household registration and place of residence are located within the same county/city and who had at least one child working away from home during the past year. (3) Migrant older adults: individuals whose place of household registration is not located in the same county/city as their current place of residence and who had at least one child working away from home during the past year.

#### 2.2.3 Moderating variable

The first group of moderating variables included Internet use status (yes/no) and duration of Internet use. Internet use status was derived from the two questions: “Do you use mobile devices, e.g. a mobile phone or tablet PC, to access the Internet?” and “Do you use a computer to access the Internet?” The answers were “yes” or “no.” If the answer to both questions was no, it indicated no Internet usage. If the answer to at least one question was yes, it indicated Internet usage. The duration of Internet use focused on usage intensity rather than simple access. Older adults were asked, “In general, how long do you access the Internet using mobile devices every day?” Responses were recorded in minutes, and Internet use duration was measured as the logarithm of this value. Furthermore, the gender of the respondents was represented as a dummy variable, with 1 indicating male and 0 indicating female.

#### 2.2.4 Control variable

We also controlled for various individual and family characteristics of the respondents, including age (in years), health status (1 = *poor*, 2 = *fair*, 3 = *good*), years of education, marital status (1 = *married*, 0 = *other*), whether the respondent had health insurance (1 = *yes*, 0 = *no*), self-assessed income status, quality of relationships with children, and geographic region (1 = *western*, 2 = *central*, 3 = *eastern*).

### 2.3 Statistical methods

We used ordinary least squares (OLS) regression models to analyze the relationship between the migration status of rural older adults, Internet use, and gender differences in their psychological well-being, employing Stata 15 for the analysis. First, we analyzed Internet use as a binary variable (yes/no). Second, we analyzed Internet use in terms of duration.

Recognizing that the decision to use the Internet among rural older adults may be influenced by individual characteristics such as their education level, age, or economic status, and the situation of other family members, it is important to note that the initial conditions of those who do not use the Internet differ from those who do. Consequently, the probabilities of Internet use status may vary, resulting in potential selection bias when directly comparing the psychological well-being of older individuals based on their Internet use. To mitigate this issue, we employed propensity score matching (PSM) to align samples of older individuals who did not use the Internet with those who did, thereby enabling a more accurate assessment of the effects associated with Internet use. First, nearest neighbor matching with a 1:4 ratio was conducted as the primary matching strategy. To assess the robustness of the results, radius matching with a caliper of 0.01 and kernel matching were subsequently applied as alternative matching methods.

Propensity scores were estimated using a logistic regression model that included all pre-treatment covariates. The matching variables comprised older adults' age group (1 = *50–59*, 2 = *60–69*, 3 = *70–79*, and 4 = *80*+ *years*), educational attainment (1 = *no schooling*, 2 = *elementary school*, 3 = *junior high school*, 4 = *high school and above*), presence of chronic diseases in the past 6 months (1 = *yes*, 0 = *no*), annual household income, number of family members working away from home, total household size, and interviewer-rated respondent cognitive ability (from 1 = *very poor* to 7 = *very good*). Matching was restricted to the region of common support. Covariate balance before and after matching was evaluated using standardized mean differences (SMDs). OLS regression analyses were subsequently conducted on the matched samples.

## 3 Results

### 3.1 Descriptive statistics

We began our analysis by computing descriptive statistics for several variables. Table 1 presents the descriptive statistics for the sample, disaggregated by gender. The average depression score among rural older adults was 34.27, which was below the midpoint of the interval. Notably, women had higher depression scores than their male counterparts (35.60 vs. 33.00, *p* < 0.001). In terms of migration status, the proportion of left-behind older individuals was 21.18%, with a higher prevalence among left-behind women than men (24.03% vs. 18.45%, *p* < 0.001). The percentage of older migrants was < 6%, and migrant women were significantly underrepresented compared with migrant men (2.62% vs. 8.74%, *p* < 0.001). Additionally, the proportion of rural older adults using the Internet was 27.82%, with female users lagging behind their male counterparts (24.25% vs. 31.25%, *p* < 0.001). Men spent approximately six more minutes per day using the Internet than women (22.41 vs. 28.58, *p* < 0.001). Furthermore, women reported lower levels of self-rated health, fewer years of education, and lower marriage rates than men (*p* < 0.001), as well as a lower proportion of health insurance coverage (*p* < 0.01).

**Table 1:** Descriptive statistics.

**Variable**	**Women**	**Men**	**Total**	***T* test/χ^2^ test**
**Dependent variable**				
Depression, *M* (*SD*)	35.60 (9.26)	33.00 (8.62)	34.27 (9.03)	11.646^***^
**Independent variables**				
**Migration status, %**				
Older adults without child migration	73.36	72.82	73.08	128.00^***^
Left-behind older adults	24.03	18.45	21.18	
Older migrants	2.62	8.74	5.74	
**Internet use status, %**				
Yes	24.25	31.25	27.82	39.02^***^
No	75.75	68.75	72.18	
Duration of internet use, *M* (*SD*)	22.41 (59.19)	28.58 (65.87)	25.57 (62.76)	−3.94^***^
**Control variables**				
Age, *M* (*SD*)	60.76 (8.07)	61.04 (8.23)	60.90 (8.15)	−1.40
**Health condition, %**				
Good	55.68	66.10	61.00	104.57^***^
Fair	14.71	15.00	14.86	
Poor	29.61	18.90	24.14	
Years of education, *M* (*SD*)	4.04 (4.09)	6.56 (3.83)	5.33 (4.16)	−25.40^***^
**Marital status, %**				
Married	86.06	90.26	88.20	27.15^***^
Other	13.94	9.74	11.80	
**Health insurance, %**				
Yes	90.65	92.76	91.73	9.40^**^
No	9.35	7.24	8.27	
Self-assessed income status, *M* (*SD*)^a^	3.12 (1.19)	3.08 (1.15)	3.10 (1.17)	1.49
Relationship with children, *M* (*SD*)^b^	4.70 (6.21)	4.62 (5.99)	4.66 (6.10)	0.56
**Region, %**				
Western	42.21	40.84	41.51	3.71
Central	29.20	28.38	28.78	
Eastern	28.59	30.79	29.71	
Observations (*N*)	3134	3274	6408	

^**^p < 0.01, ^***^p < 0.001.

^a^For self-assessed income status, the respondents were asked “What is your relative income level in your local area?” The responses (from very low to very high) were rated on a scale of 1–5.

^b^For quality of relationships with children, the respondent was asked about how the relationship was between him/her and each of his/her children in the past 6 months. The response was rated using a 5-point frequency response scale (1 = not close at all, 2 = not very close, 3 = fair, 4 = close, and 5= very close). The relationship state scores for each child were added together. The total score for this question ranged from 1 to 38, with higher scores indicating better quality of relationships with children.

### 3.2 Results of PSM method

We then conducted propensity score matching in order to select demographically comparable subsamples. The results of the nearest neighbor matching method concerning Internet use status are presented in Table 2. The balance test conducted after matching revealed that the characteristics of the two groups regarding Internet use status were highly comparable, indicating a satisfactory balance between the samples. For example, as shown in Table 3, the age distribution was well balanced across the four age groups (50–59, 60–69, 70–79, and 80+ years) after matching, with all absolute SMDs below the conventional 10% threshold.

**Table 2:** Results for the three PSM methods.

**PSM methods**	**Treated (*n*)**	**Control (*n*)**	**ATT**	**SE**	***T***
Nearest neighbor matching	1,777	4,576	1.30^*^	0.61	2.14
Radius matching	1,781	4,620	1.06^*^	0.51	1.97
Kernel matching	1,783	4,625	1.62^*^	0.28	2.24

The full results are available upon request. ATT, average treatment effect on the treated. ^*^p < 0.05.

**Table 3:** Covariate balance for age groups among rural adults before and after matching (SMD, %).

**Age group**	**Unmatched**	**Nearest neighbor matching**	**Radius matching**	**Kernel matching**
50–59	80.9	2.1	0.9	9.3
60–69	−41.4	−2.6	−0.8	−6.6
70–79	−53.8	−0.7	−0.3	−7.9
80 years and above	−19.5	−0.2	−0.2	−2.6

SMD refers to standardized mean differences. Absolute SMD values below 10% indicate adequate covariate balance.

The results of the OLS regression models following propensity score matching are presented in Table 4. For Models 1a−1c, effect sizes were interpreted using a consistent CES-D scale. In population-based research on older adults, differences of around one point on the CES-D scale are commonly considered meaningful, as they reflect perceptible changes in the frequency or intensity of depressive symptoms rather than random fluctuation. Accordingly, coefficients of approximately half a point or more are interpreted as substantively relevant, while differences exceeding one point are viewed as practically significant. First, we examined the results of nearest neighbor matching. Model 1a indicates that, after controlling for other variables, rural left-behind older individuals and older migrants exhibited significantly more severe depressive symptoms and poorer mental health than older individuals whose children had not migrated (left-behind older individuals: β = 0.465, *p* < 0.05; older migrants: β = 0.965, *p* < 0.05). In substantive terms, these coefficients correspond to increases of approximately half a point to one point on the CES-D scale, which are commonly regarded as meaningful differences in emotional well-being in population-based studies of older adults.

**Table 4:** OLS model analyzing gender differences in migration status, Internet use status, and mental health among rural older adults (Nearest neighbor matching, 95% confidence interval, standard errors).

**Variable**	**Model 1a**	**Model 1b**	**Model 1c**
**Migration status (older adults without child migration** = **1)**			
Left-behind older adults	0.465^*^ (−0.047 to 0.977) (0.261)	0.267 (−0.334 to 0.868) (0.307)	0.172 (−0.628 to 0.972) (0.408)
Older migrants	0.965^*^ (0.057 to 1.873) (0.463)	0.268 (−0.976 to 1.511) (0.634)	−0.236 (−2.675 to 2.204) (1.244)
Internet use status (no = 0)	−0.679^**^ (−1.182 to −0.175) (0.257)	−0.954^**^ (−1.540 to −0.368) (0.299)	−0.936^*^ (−1.775 to −0.098) (0.428)
Male (female = 0)	−1.552^***^ (−1.991 to −1.113) (0.224)	−1.550^***^ (−1.89 to −1.111) (0.224)	−1.470^***^ (−2.047 to −0.893) (0.294)
**Older adults without child migration** × **Internet use status**			
Left-behind older adults × Yes		0.713^†^ (−0.414 to 1.840) (0.575)	1.163 (−0.428 to 2.753) (0.811)
Older migrants × Yes		1.528^*^ (−0.262 to 3.318) (0.913)	5.022^**^ (1.306 to 8.738) (1.896)
**Older adults without child migration** × **Internet use status**			
× **Male**			
Left-behind older adults × Yes × Male			−0.937^†^ (−3.198 to 1.325) (1.154)
Older migrants × Yes × Male			−4.392^*^ (−8.644 to −0.140) (2.169)
Age	−0.031 (−0.070 to 0.009) (0.020)	−0.032 (−0.071 to 0.007) (0.020)	−0.033 (−0.072 to 0.007) (0.020)
**Health condition (poor** = **1)**			
Good	−6.326^***^ (−6.833 to −5.818) (0.259)	−6.321^***^ (−6.829 to −5.813) (0.259)	−6.332^***^ (−6.840 to −5.824) (0.259)
Fair	−4.437^***^ (−5.116 to −3.757) (0.346)	−4.429^***^ (−5. 109 to −3.750) (0.347)	−4.443^***^ (−5.123 to −3.764) (0.347)
Years of education	−0.117^***^ (−0.172 to −0.061) (0.028)	−0.116^***^ (−0.172 to −0.060) (0.028)	−0.118^***^ (−0.174 to −0.062) (0.029)
Marital status (other = 0)	−2.997^***^ (−3.658 to −2.336) (0.337)	−3.011^***^ (−3.672 to −2.350) (0.337)	−3.030^***^ (−3.691 to −2.368) (0.338)
Health insurance (no = 0)	−1.595^***^ (−2.347 to −0.844) (0.383)	−1.601^***^ (−2.352 to −0.849) (0.383)	−1.601^***^ (−2.353 to −0.850) (0.383)
Self-assessed income status	−0.747^***^ (−0.926 to −0.567) (0.092)	−0.746^***^ (−0.926 to −0.566) (0.092)	−0.745^***^ (−0.925 to −0.565) (0.092)
Relationship with children	−0.047^†^ (−0.096 to 0.002) (0.025)	−0.047^†^ (−0.096 to 0.002) (0.025)	−0.047^†^ (−0.096 to 0.002) (0.025)
**Region (western** = **1)**			
Eastern	−1.749^***^ (−2.254 to −1.244) (0.258)	−1.750^***^ (−2.255 to −1.245) (0.258)	−1.756^***^ (−2.261 to −1.251) (0.258)
Central	−1.056^***^ (−1.595 to −0.516) (0.275)	−1.057^***^ (−1.596 to −0.517) (0.275)	−1.067^***^ (−1.607 to −0.527) (0.276)
Constant	49.769^***^ (47.142 to 52.397) (1.340)	49.942^***^ (47.309 to 52.575) (1.343)	49.964^***^ (47.323 to 52.606) (1.347)
*R* ^2^	0.156	0.156	0.157
*N*	6,353	6,353	6,353

^†^*p* < 0.1, ^*^p < 0.05, ^**^p < 0.01, ^***^p < 0.001.

We also found that older individuals who used the Internet had less severe depressive symptoms than those who did not, and men had fewer depressive symptoms than their female counterparts (Internet use status: β = 0.679, *p* < 0.01; gender: β = 1.552, *p* < 0.001). Taken together, coefficients in Model 1a indicate changes of approximately one half to one and a half points on the CES-D scale, which are generally regarded as meaningful differences in mental health rather than trivial fluctuations. Overall, these findings suggest that Internet use had a statistically positive effect on the mental health of rural older adults, and that rural older men experienced better mental health outcomes than rural older women.

Additionally, significant regional disparities in depressive symptoms were observed. Compared with the western region, older adults residing in the eastern region and central region exhibited significantly lower levels of depressive symptoms (eastern region: β = 1.749, *p* < 0.001; central region: β = 1.056, *p* < 0.001). The larger magnitude of the coefficient for the eastern region indicates a more pronounced reduction in depressive symptoms relative to the central region, suggesting a clear regional gradient.

The term for interaction between migration status and Internet use status in Model 1b indicates that, after controlling for other variables, older migrants who used the Internet had more pronounced depressive symptoms than older individuals whose children had not migrated (β = 1.528, *p* < 0.05). Substantively, this coefficient suggests that the depressive-symptom-reducing effect of Internet use status among older migrants was approximately 1.5 CES-D points weaker than that observed among their counterparts whose children had not migrated. Given that even a one-point difference on the CES-D scale is often considered meaningful in population-based mental health research, this result reflects a non-trivial moderation effect of migration status on the mental health implications of Internet use status. This finding suggests that the positive impact of Internet use status on the mental health of older migrants was less pronounced than that observed among their counterparts whose children had not migrated.

A similar but weaker pattern was observed for left-behind older adults. Although the interaction between Internet use status and left-behind status reached only marginal significance (β = 0.713, *p* < 0.1), the positive coefficient indicates that the mental health benefits of Internet use status were also attenuated in this group. In magnitude, the interaction effect for left-behind older adults was approximately 0.7 CES-D points, which is notably smaller than that observed for older migrants (β = 1.528). This finding indicates that both left-behind older adults and older migrants who used the Internet exhibited more pronounced depressive symptoms than older adults whose children had not left. In other words, the positive impact of Internet use status on the mental health of rural older adults was strongest for older adults whose children had not migrated and weakest for older migrants. Therefore, these findings provide support for ***H1***.

**Figure 1:**
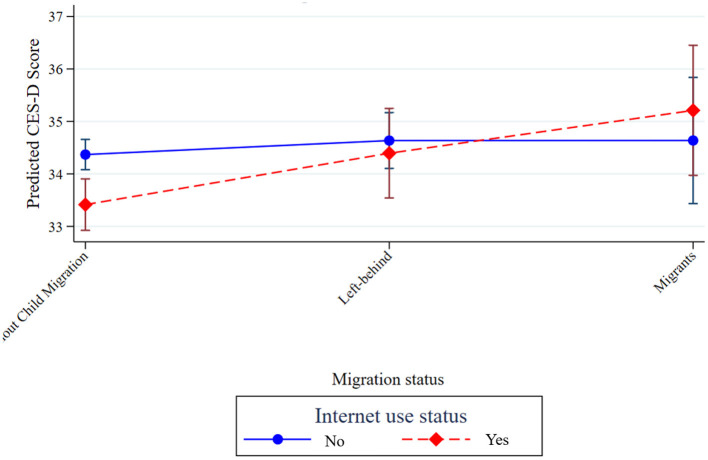
Interaction between migration status and Internet use status (Nearest neighbor matching, source: CFPS2020).

Figure 1 presents the predictive margins of depressive symptoms (CES-D scores) across migration status, stratified by Internet use status. The interaction plot indicates that the association between migration status and depressive symptoms differs by Internet use status. Among older adults who do not use the Internet, depressive symptoms differ only modestly across migration statuses. In contrast, among Internet users, depressive symptoms differ significantly among migration statuses, suggesting that Internet use status modifies the relationship between migration status and mental health. While Internet use status is generally associated with a lower average level of depressive symptoms, the interaction plot suggests that among older adults experiencing migration-related disruptions, Internet use status may not fully offset the adverse mental health consequences and may even coincide with a higher level of depressive symptoms.

**Figure 2:**
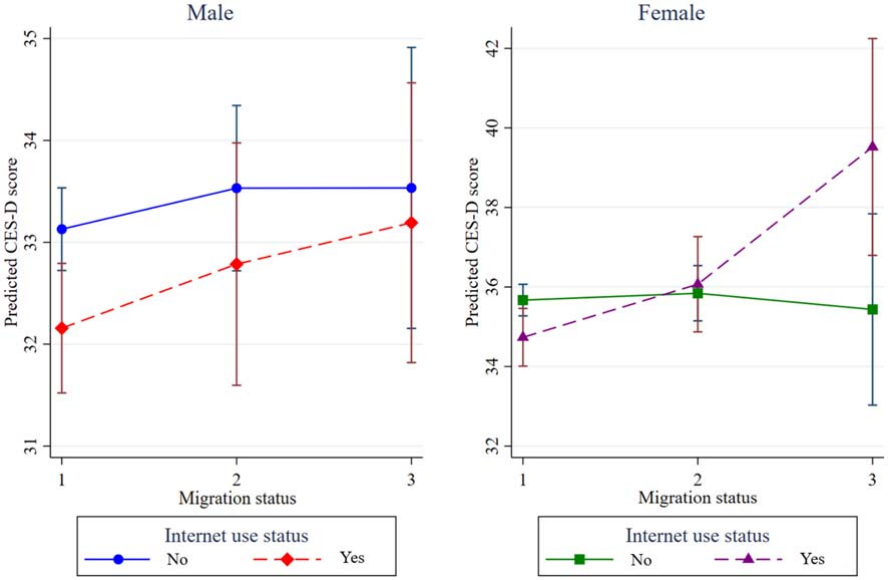
Interaction among migration status, Internet use status, and gender (Nearest neighbor matching, source: CFPS2020).

Figure 2 further illustrates the three-way interaction among migration status, Internet use status, and gender by presenting predictive margins separately for men and women. Among older men, Internet use status is consistently associated with lower predicted CES-D scores across migration statuses, and the gap between Internet users and non-users remains relatively stable as migration status changes. This pattern suggests that Internet use status provides a protective mental health benefit for men, including those experiencing migration-related disruptions.

In contrast, among older women, the pattern differs markedly. While Internet use status is associated with slightly lower depressive symptoms among women whose children had not migrated, predicted CES-D scores among female Internet users increase sharply with migration status. In particular, among older migrant women, Internet users exhibit substantially higher levels of predicted depressive symptoms than their non-Internet-using counterparts. This divergence visually demonstrates that the mental health benefits of Internet use status are considerably weaker for women and may even reverse among those experiencing migration-related stress.

Consistent with this visual pattern, the term for the interaction between migration status, Internet use status, and gender in Model 1c indicates that, after controlling for other variables, the depressive-symptom-reducing effect of Internet use status was significantly stronger among older migrant men than among older migrant women (β = −4.392, *p* < 0.05). Substantively, this coefficient implies that the mental health benefit of Internet use status for older migrant men is more than four CES-D points greater than that for older migrant women. Given that changes of one to two points on the CES-D scale are commonly regarded as meaningful in population-based mental health research, this gender difference represents a strong and practically significant effect. Therefore, these findings provide support for ***H2***.

A similar but more modest gender difference was observed among left-behind older adults. The term for the interaction between Internet use status and gender was marginally significant (β = −0.937, *p* < 0.1), indicating that the depressive-symptom-reducing effect of Internet use status was approximately one CES-D point greater for left-behind older men than for left-behind older women. As shown in Figure 2, the trajectories of depressive symptoms for male Internet users and non-users remain relatively close among left-behind older adults. Whereas among women, Internet use is associated with higher predicted CES-D scores as migration status shifts from non-migrant to left-behind and then to migrant status. Although smaller in magnitude than the effect observed among older migrants, this pattern suggests a modest but non-negligible gender difference in the mental health returns to Internet use status within left-behind contexts. Therefore, these findings provide support for ***H3***.

Overall, these interaction effects are not only statistically significant but also substantively meaningful. In particular, the gender difference observed among older migrants (clearly illustrated in Figure 2) underscores the importance of considering intersecting social positions, such as migration status and gender, when evaluating the mental health implications of Internet use status in later life.

### 3.3 Robustness check

We tested additional PSM methods for robustness checks. These methods include radius matching and kernel matching. The results of the matching method concerning Internet use status are presented in Table 2. The covariate balance for age groups before and after matching is presented in Table 3. Overall, the new results from Tables 5, 6 confirm the robustness of the nearest neighbor matching method.

**Table 5:** OLS model analyzing gender differences in migration status, Internet use status, and mental health among rural older adults (Radius matching, 95% confidence interval, standard errors).

**Variable**	**Model 2a**	**Model 2b**	**Model 2c**
**Migration status (older adults without child migration** = **1)**			
Left-behind older adults	0.486^*^ (−0.080 to 0.955) (0.264)	0.262 (−0.338 to 0.863) (0.306)	0.166 (−0.632 to 0.965) (0.407)
Older migrants	0.994^*^ (−0.016 to 1.830) (0.471)	0.272 (−0.975 to 1.518) (0.636)	−0.209 (−2.654 to 2.236) (1.247)
Internet use status (no = 0)	−0.659^*^ (−1.170 to −0.148) (0.261)	−0.962^**^ (−1.513 to −0.322) (0.304)	−0.863^*^ (−1.718 to −0.008) (0.436)
Male (female = 0)	−1.532^***^ (−1.974 to −1.089) (0.226)	−1.523^***^ (−1.971 to −1.086) (0.226)	−1.447^***^ (−2.022 to −0.871) (0.294)
**Older adults without child migration** × **Internet use status**			
Left-behind older adults × Yes		0.800^†^ (−0.490 to 1.825) (0.591)	0.986 (−0.628 to 2.601) (0.824)
Older migrants × Yes		1.584^*^ (−0.387 to 3.257) (0.929)	5.026^**^ (1.269 to 8.783) (1.917)
**Older adults without child migration** × **Internet use status**			
× **Male**			
Left-behind older adults × Yes × Male			−0.850^†^ (−3.032 to 1.617) (1.186)
Older migrants × Yes × Male			−4.521^*^ (−8.829 to −0.213) (2.198)
Control variable	Yes	Yes	Yes
*R* ^2^	0.155	0.155	0.156
*N*	6,401	6,401	6,401

^†^*p* < 0.1, ^*^p < 0.05, ^**^p < 0.01, ^***^p < 0.001.

**Table 6:** OLS model analyzing gender differences in migration status, Internet use status, and mental health among rural older adults (Kernel matching, 95% confidence interval, standard errors).

**Variable**	**Model 3a**	**Model 3b**	**Model 3c**
**Migration status (older adults without child migration** = **1)**			
Left-behind older adults	0.486^*^ (−0.025 to 0.996) (0.260)	0.264 (−0.335 to 0.863) (0.305)	0.168 (−0.629 to 0.950) (0.407)
Older migrants	0.994^*^ (0.086 to 1.901) (0.463)	0.272 (−0.972 to 1.516) (0.634)	−0.207 (−2.647 to 2.232) (1.244)
Internet use status (no = 0)	−0.663^**^ (−1.166 to −0.160) (0.257)	−0.962^**^ (−1.548 to −0.377) (0.299)	−0.935^*^ (−1.772 to −0.982) (0.427)
Male (female = 0)	−1.526^***^ (−1.963 to −1.089) (0.223)	−1.523^***^ (−1.961 to −1.086) (0.223)	−1.447^***^ (−2.021 to −0.873) (0.293)
**Older adults without child migration** × **Internet use status**			
Left-behind older adults × Yes		0.800^†^ (−0.324 to 1.924) (0.573)	1.206 (−0.381 to 2.792) (0.809)
Older migrants × Yes		1.584^*^ (−0.204 to 3.712) (0.912)	4.994^**^ (1.278 to 8.710) (1.896)
**Older adults without child migration** × **Internet use status**			
× **Male**			
Left-behind older adults × Yes × Male			−0.850^+^ (−3.105 to 1.406) (1.151)
Older migrants × Yes × Male			−4.275^*^ (−8.525 to −0.024) (2.168)
Control variable	Yes	Yes	Yes
*R* ^2^	0.155	0.155	0.156
*N*	6,408	6,408	6,408

^†^*p* < 0.1, ^*^p < 0.05, ^**^p < 0.01, ^***^p < 0.001.

### 3.4 Further discussion: duration of Internet use

Table 7 presents OLS estimates examining gender differences in the associations between migration status, duration of Internet use, and depressive symptoms among rural older adults. Model 4a reports baseline estimates, while Models 4b and 4c sequentially introduce two-way and three-way interaction terms.

**Table 7:** OLS model analyzing gender differences in migration status, duration of Internet use, and mental health among rural older adults (95% confidence interval, standard errors).

**Variable**	**Model 4a**	**Model 4b**	**Model 4c**
**Migration status (older adults without child migration** = **0)**			
Left-behind older adults	0.486^†^ (−0.024 to 0.997) (0.260)	0.284 (−0.305 to 0.872) (0.300)	0.172 (−0.614 to 0.958) (0.401)
Older migrants	1.143^*^ (0.236 to 2.051) (0.463)	0.502 (−0.704 to 1.709) (0.616)	0.204 (−2.149 to 2.557) (1.200)
Duration of internet use	−0.128^*^ (−0.234 to 0.002) (0.060)	−0.183^**^ (−0.319 to 0.046) (0.070)	−0.153 (−0.349 to 0.043) (0.100)
Male (female = 0)	−1.540^***^ (−1.976 to −1.104) (0.222)	−1.538^***^ (−1.973 to −1.102) (0.222)	−1.429^***^ (−1.994 to −0.864) (0.288)
**Older adults without child migration** × **Duration of Internet use**			
Left-behind older adults × Duration of Internet use		0.183 (−0.448 to 0.807) (0.135)	0.276 (−0.096 to 0. 648) (0.190)
Older migrants × Duration of Internet use		0.346^†^ (−0.064 to 0.755) (0.209)	1.042^*^ (0.181 to 1.903) (0.439)
**Older adults without child migration** × **Duration of Internet** × **Male use**			
Left-behind older adults × Duration of Internet use × Male			−0.205 (−0.737 to 0.326) (0.271)
Older migrants × Duration of Internet use × Male			−0.849^†^ (−1.831 to 0. 132) (0.501)
Control variable	Yes	Yes	Yes
Constant	45.429^***^ (44.370 to 46.487) (0.540)	45.515^***^ (44.453 to 46.577) (0.542)	45.510^***^ (44.433 to 46.587) (0.549)
*R* ^2^	0.158	0.158	0.159
*N*	6,408	6,408	6,408

^†^*p* < 0.1, ^*^p < 0.05, ^**^p < 0.01, ^***^p < 0.001.

Model 4a shows that, a longer duration of Internet use was significantly associated with lower CES-D scores (β = −0.128, *p* < 0.05), indicating that spending more time online was linked to better mental health outcomes on average.

Model 4b introduces interaction terms between migration status and duration of Internet use to assess whether the mental health effects of Internet usage time vary across migration contexts. The interaction between duration of Internet usage and left-behind status was positive but not statistically significant. In contrast, the interaction between duration of Internet use and older migrant status was positive and marginally significant (β = 0.346, *p* < 0.1), indicating that the mental health benefits associated with longer Internet use are weaker among older migrants than among those whose children had not migrated.

Model 4c further incorporates terms for the three-way interaction among migration status, duration of Internet use, and gender. The interaction term for older migrants was negative and marginally significant (β = −0.849, *p* < 0.1), indicating that the attenuating effect of longer Internet use on depressive symptoms among older migrants was less pronounced for women than for men. In substantive terms, older migrant men derived greater mental health benefits from longer periods of Internet use than older migrant women. In contrast, the three-way interaction involving left-behind older adults was small and not statistically significant, suggesting limited gender difference in the mental health effects of Internet use duration within left-behind contexts.

Taken together, the results from Table 7 indicate that the association between duration of Internet use and mental health is contingent on both migration status and gender. These findings show some consistency with earlier interaction patterns based on Internet use status and correspond in part to those illustrated in Figure 2, indicating that the psychological implications of Internet engagement in later life differ across intersecting social positions.

## 4 Conclusion

In our study, we employed the theoretical framework of social capital to examine the experiences of older migrants, left-behind older adults, and older adults whose children had not migrated from rural areas. We specifically investigating whether there were gender differences in how Internet use affects the mental health of these groups. Our study found the following. First, left-behind older adults and older migrants in rural areas had more pronounced depressive symptoms and poorer mental health than older adults whose children had not migrated. Second, the positive impact of Internet use (measured by Internet use status (yes/no) and usage duration) on the mental health of rural older adults varied across different migration statuses. The effects were least pronounced for migrant older adults, followed by left-behind older adults, and most pronounced for older individuals whose children had not migrated. Third, the positive impact of Internet use status on the mental health of migrant and left-behind older women was significantly lower than that observed for their male counterparts. Finally, given the cross-sectional and observational design of this study, the findings should be interpreted as associational rather than causal.

## 5 Discussion and recommendations

Our findings revealed that the beneficial effects of Internet use on the mental health of rural older men were more pronounced than those for rural older women, which contrasts with the results of Yang et al. (55). Their analysis of individuals aged 60 and above, using CFPS data from 2016 to 2020, concluded that the positive impact of the Internet on older women exceeded that on men. This discrepancy may stem from their finding that the Internet primarily benefits older individuals living in urban areas rather than those living in rural areas, whereas our study focuses specifically on rural older populations. Rural older adults, particularly women, who often rely more on family support networks for their well-being than on Internet usage, exhibit lower levels of Internet acceptance and usage than their urban counterparts.

From a social capital perspective, the nonsignificant interaction between left-behind status and the duration of Internet use suggests that extended online engagement does not substantially alter the structure or accessibility of social resources among left-behind older adults relative to those whose children had not migrated. While Internet access may facilitate the maintenance of existing bonding ties, additional time spent online appears insufficient to generate differentiated mental health benefits across these relatively similar family contexts. The non-significant three-way interaction involving left-behind status, Internet use duration, and gender indicates that prolonged online engagement does not produce substantial gender differences in mental health benefits within left-behind contexts. From a social capital perspective, this finding suggests that Internet use among left-behind older adults primarily reinforces existing bonding ties in a largely gender-neutral manner, limiting the emergence of gender differences in mental health outcomes. These findings suggest that the mental health implications of Internet use in later life are not primarily driven by the duration of online engagement. Instead, they depend more on whether Internet use enables access to, or the restructuring of, social capital under conditions of social disruption.

From a social capital perspective, the finding that Internet use provides fewer mental health benefits for women, especially migrants, reflects gendered processes in the accumulation and conversion of social capital. Internet use does not uniformly translate into psychological benefits; its effects depend on whether online engagement facilitates access to emotionally supportive and meaningful social ties. Older women may face structural constraints that limit their ability to mobilize online resources into mental health protective social capital. This pattern likely results from the combined effects of lower digital literacy, a stronger reliance on traditional family support which is disrupted by migration, and gender differences in patterns of online social capital. Social gender-role theory suggests that older women's psychological well-being is more strongly tied to family-based, face-to-face support (48), making Internet-mediated interactions less effective in compensating for the disruption of core social ties caused by migration. These mechanisms are further amplified among older migrant women, who occupy a doubly disadvantaged position in which migration weakens traditional sources of support while digital technologies offer limited opportunities to reconstruct emotionally supportive networks. Overall, these findings suggest that the mental health implications of Internet use in later life are more closely tied to the social functions that Internet use serves in gendered and migration-specific contexts than to the duration of online engagement.

Taken together, our findings advance understanding of how Internet use, migration status, and gender intersect to shape mental health among rural older adults through gendered processes of social capital. However, these interpretations should be viewed cautiously. The cross-sectional and observational design of this study precludes causal inference. Accordingly, the results should be interpreted as correlational rather than causal. Future research using longitudinal or quasi-experimental designs is needed to clarify causal pathways linking Internet use, migration, gender, and mental health in later life.

The positive impact of the Internet on the mental health of migrant older adults varies by gender and urban–rural backgrounds. This underscores the need for policies and interventions that move beyond uniform approaches and address gendered and migration-specific digital inequalities. Policy efforts should build on the central role that family relationships play in the well-being of rural older women, particularly migrants. Given that family connections are the primary motivation for ICT use among older migrants (56), policies should seek to strengthen the capacity of Internet use to sustain and enhance family-based, emotionally supportive interactions across geographic distance. At the same time, policy interventions should place greater emphasis on addressing the structural conditions that shape older women's ability to benefit from digital technologies. This includes reducing digital inequalities through improved access to age-friendly digital infrastructure, enhancing digital literacy, and providing supportive community-based resources that enable older migrant women to translate online family contact into meaningful social support and mental health benefits.

A central policy priority should be the reduction of digital inequalities through targeted digital literacy interventions. Older migrant women from rural areas often lack nearby family members who can provide informal assistance with technology use, placing them at a disadvantage in acquiring and maintaining digital skills. Community-based, one-on-one or small-group digital literacy programs (delivered through village committees, community centers, or social work agencies) can help address this gap by providing sustained, personalized support tailored to older women with lower socioeconomic status (42, 57). Such programs should emphasize practical, socially oriented Internet use that supports relationship maintenance and emotional well-being, rather than purely technical skills.

Importantly, improving digital skills alone may be insufficient if older adults lack the motivation or confidence to engage with digital technologies (58). Therefore, complementary measures (such as integrating digital training with community activities, peer support networks, or family-mediated participation) are essential to enhance engagement and learning outcomes. For older migrant women from rural areas, combining digital literacy training with broader community support structures can help ensure that Internet use becomes a viable resource for rebuilding social capital and improving mental health.

## 6 Limitations

This study has several limitations. First, because it relied on cross-sectional data, our findings could only demonstrate correlations between variables rather than establishing cause-and-effect relationships. Consequently, the results should be interpreted with caution, and future research should use longitudinal data to further investigate the causal relationships between these variables. Second, although we considered the selectivity of rural older adults' migration, our methodological approach could not fully address the issue of sample selection due to unobserved heterogeneity. In particular, unmeasured factors such as motivations for migration, digital skills, and cognitive ability may jointly influence migration status, Internet use, and mental health outcomes, potentially biasing the estimated effects. Moreover, older migrants are a heterogeneous group, and differences in migration motivations (e.g., caregiving responsibilities, access to health care, or employment) may shape their social networks and stress levels in distinct ways. The inability to capture such within-group heterogeneity represents an additional limitation and warrants further investigation in future research. Finally, although this study employed two indicators to measure Internet use—whether older adults used the Internet and the duration of Internet use—the measurement of digital engagement remains limited. These indicators do not reflect qualitative dimensions such as Internet use frequency, the specific types of online activities (e.g., communication vs. entertainment) engaged in, digital skills, or digital self-efficacy. These unobserved dimensions may partly explain the observed gender differences and heterogeneous effects across migration groups. Future research should adopt more comprehensive measures of Internet use to better capture the multifaceted nature of digital engagement.

## Data Availability

Publicly available datasets were analyzed in this study. This data can be found here: https://www.isss.pku.edu.cn/cfps/en/
